# Section traumatique de l'utérus non gravide chez une accidentée de voie publique: à propos d'un cas

**DOI:** 10.11604/pamj.2015.20.173.5788

**Published:** 2015-02-24

**Authors:** Sangwa Milindi Cédrick, Kitembo Feruzi Marius, Kakinga Zabibu Mireille, Mukonda Sompo Nelly, Cham Lubamba Chamy, Kabamge Numbi, Jean Felix Mutomb

**Affiliations:** 1 Hôpital Général Provincial de Référence Jason Sendwe, Faculté de Médecine, Université de Lubumbashi, Lubumbashi, République Démocratique de Congo; 2 Institut de Recherche en Sciences de la Santé, antenne de Lubumbashi, République Démocratique du Congo

**Keywords:** Fracture, bassin, hemopéritoine, utérus, Fracture, pelvis, haemoperitoneum, uterus

## Abstract

Dans cet article les auteurs rapportent un rare cas de section traumatique d'un utérus non gravide, chez une accidentée de voie publique reçu dans un tableau d'hemopéritoine aux services des urgences de l'hôpital General Provincial de Reference Jason Sendwe.

## Introduction

Une rupture utérine correspond à une solution de continuité non chirurgicale de l'utérus gravide atteignant le corps ou le segment inférieur de l'utérus au cours de la grossesse ou du travail. Parmi les étiologies, les traumatismes représentent moins de 1% des cas [1]. L'atte inte traumatique de l'utérus est en rapport avec sa position abdominale du fait de la grossesse. Ceci expliquerait que la plupart des ruptures utérines par accident de voie publique surviennent surtout au 3^ème^ trimestre [2, 3]. En dehors de la grossesse, l'atteinte de l'utérus lors d'une contusion abdominale est exceptionnelle du fait de sa localisation pelvienne. Lorsque cette contusion est associée à une fracture du bassin, les lésions vasculaires, neurologiques et même urogénitales peuvent survenir [4]. Cependant une section traumatique et isolée d'un utérus non gravide dans les mêmes circonstances, mérite d’être évoquée.

## Patient et observation

**Figure 1:**
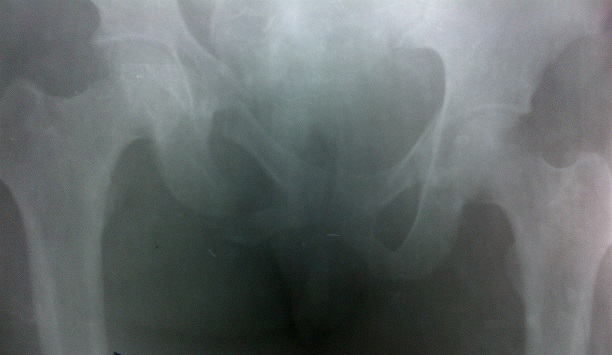
Fracture du bassin (face) de type C selon la classification de Tile

**Figure 2:**
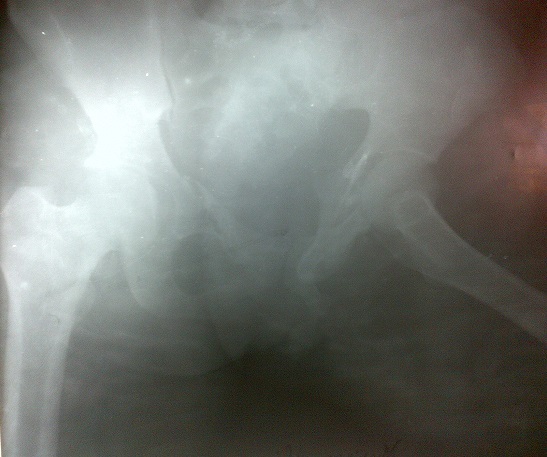
Fracture du bassin (profil) de type C selon la classification de Tile

**Figure 3:**
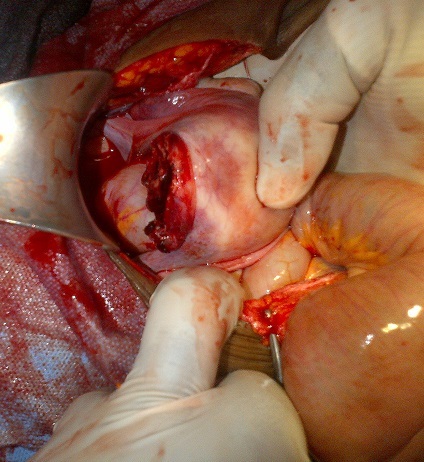
Section complète transversale de l'utérus séparé de son col au niveau de l'isthme

**Figure 4:**
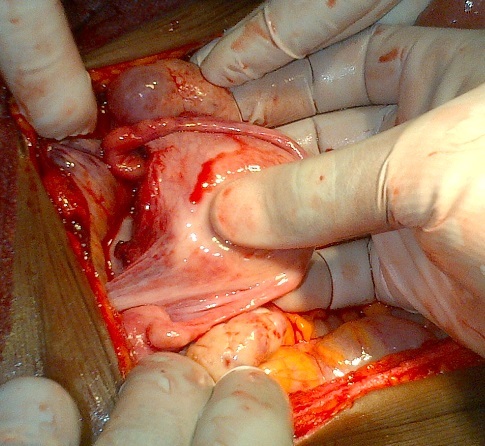
Utérus soutenu par les ligaments rond et utéro-sacrés

**Figure 5:**
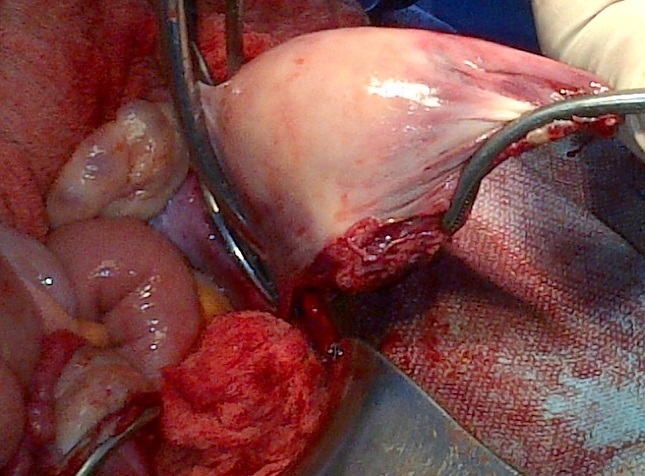
Hystérectomie subtotale

Madame X âgée de 25 ans, P1G1A0D0 (P: Parité, G: Gesteté, A: Avortement, D: Décès), a été reçu en date du 02 septembre 2014 aux services des urgences de l'hôpital Général de Référence Jason Sendwe pour douleur abdominale, et impotence fonctionnelle de la hanche gauche. Dans l'histoire, la patiente serait victime d'un accident de voies public au cours duquel une voiture roulant à vive allure l'aurait percuté à la hanche droite et projetée contre un mur de clôture. Les suites post traumatique étaient marquées par une vive douleur de la hanche avec impotence fonctionnelle rendant la marche impossible. La victime a été acheminée à un centre proche de sa résidence pour des soins palliatifs. La persistance des signes ci- haut cités et l'installation d'une vive douleur abdominale a motivé 48 heures après un transfert à l'HGR Sendwe pour une meilleur prise en charge. A notre examen clinique, la patiente avait présenté un état général altéré par l'asthénie. Les signes vitaux étaient altérés avec un effondrement de la tension artérielle. Les conjonctives étaient pâles, la langue sèche et l'abdomen contracturé. Une ponction transpariétale a ramené du sang non coagulable. Le diagnostic de contusion abdominale compliquée d'hemopéritoine a été retenu. La radiographie faite en urgence a révélé une fracture instable du bassin de type C selon la classification de Tile (Figure 1, Figure 2), l’échographie abdominale faite le même jour, a confirmé la présence d'un épanchement péritonéal sans toutefois en préciser l'origine vue que les viscères pleins étaient apparemment intacts. La laparotomie exploratrice réalisée en urgence deux heures après l'admission de la victime a mise en évidence un épanchement sanguin important (2000 cc de sang). Le foie et la rate étaient intacts. Nous avons noté dans la cavité pelvienne un utérus non gravide sectionné transversalement et qui était complètement séparé de son col au niveau de l'isthme (Figure 3). L'utérus ne tenait que par les ligaments utérosacrés, les ligaments ronds et les annexes (trompes et ovaires) (Figure 4). Nous avons également constaté une déchirure de la séreuse pariétale le long du ligament rond sans mettre mettre en évidence une plaie dont le trajet ferait communiquer la cavité pelvienne avec le foyer fracturaire du bassin. La vessie a présenté une petite zone de contusion sur sa paroi supérieure. Le rectum ainsi que le promontoire étaient sains. La sonde vésicale placée avant l'intervention a ramené des urines claires (non hématiques). Le début d'infarcisèment de l'utérus amputé de son col (hystérectomie subtotale traumatique) a justifié son ablation après consentement du mari Les annexes ont été conservées (Figure 5). L'hémostase a été assurée au niveau du moignon du col utérin. La paroi abdominale a été réfectionnée plan par plan après avoir nettoyé et asséché la cavité péritonéale. Dans les suites post opératoires, la patiente a présenté une bonne évolution clinique.

## Discussion

A l′occasion d′un traumatisme du bassin, l′éventualité d′une rupture d'un utérus non gravide est exceptionnelle, mais il faudra y penser. En effet, comme pour tous les organes intra pelviens (vessie, rectum), la proximité de l'utérus par rapport au bassin osseux le rend vulnérable en cas de fracture osseuse, a fortiori lorsque cette dernière s'accompagne d'un déplacement des fragments fracturaires. Ce déplacement est en rapport avec l’énergie impliquée au cours du traumatisme. Les fractures à basses énergie, généralement des fractures isolées d'un os, sont souvent dues à des chutes à domicile fréquentes chez les personnes âgées. Par contre, les fractures du bassin à haute énergie provoquent une rupture de l'anneau pelvien et endommagent les tissus et viscères environnants. Dans 57% des cas, comme c’était le cas chez notre patiente, ces types de fractures sont consécutives à un accident de trafic routier [5]. Notre patiente a subi un choc direct sur son bassin du côté droit. Ce choc a entraîné une force de compression latérale responsable de la fracture du bassin. Le trait de fracture qui est fonction de la localisation de l'impact, pouvait témoigner de l'importance de la force en cause lors de l'accident. La fracture du bassin était double antérieure avec disjonction de la sacro-iliaque homologue (type C selon la classification de Tile) [6]. Ces lésions ont entraîné une instabilité et une déformation squelettique importante qui se sont répercutées sur l'articulation de la hanche rendant la marche de notre patiente impossible. L'hemopéritoine présenté par notre patiente, était due au saignement des artères cervico-utérines suite à la section transversale complète de l'utérus séparé de son col au niveau de l'isthme. Cette atteinte pourrait s'expliquer par un embrochage de la paroi utérine par des esquilles ou des fragments osseux acérés habituellement obtenus dans les fractures des deux branches pubiennes, les disjonctions pubiennes supérieures à 2cm, les fractures d'un cadre obturateur avec un déplacement supérieur à 1cm et les fractures unilatérales d'un cadre obturateur associées à une luxation sacro-iliaque ainsi que les importantes disjonctions pubiennes [7]. Il est cependant important de noter que l'atteinte isolée de l'utérus par rapport aux autres organes intra pelviens retrouvés intacts, constitue une situation exceptionnelle à laquelle il est difficile d'apporter une explication claire et précise quant au mécanisme, d'autant plus qu'aucune plaie n'a fait communiquer la cavité pelvienne au foyer fracturaire.

## Conclusion

L'atteinte isolée d'un utérus non gravide au cours d'une fracture du bassin est une situation exceptionnelle mais il faudra y penser.
